# Characterizing G-type antiferromagnetism quantitatively with optical second harmonic generation

**DOI:** 10.1038/s41377-025-01849-3

**Published:** 2025-04-22

**Authors:** Shuai Xu, Cheng Ma, Kui-juan Jin, Qinghua Zhang, Sisi Huang, Yiru Wang, Xu He, Jiesu Wang, Donggang Xie, Qiulin Zhang, Er-Jia Guo, Chen Ge, Can Wang, Xiulai Xu, Lin Gu, Meng He, Guozhen Yang

**Affiliations:** 1 Beijing National Laboratory for Condensed Matter Physics, Institute of Physics, Chinese Academy of Sciences, Beijing, 100190 China; 2 University of Chinese Academy of Sciences, Beijing, 100049 China; 3 Theoretical Materials Physics, Q-MAT, Université de Liège, Liège, B-4000 Belgium; 4 Beijing Academy of Quantum Information Sciences, Beijing, 100193 China; 5 State Key Laboratory for Mesoscopic Physics and Frontiers Science Center for Nano-optoelectronics, School of Physics, Peking University, Beijing, 100871 China; 6 Beijing National Center for Electron Microscopy and Laboratory of Advanced Materials, Department of Materials Science and Engineering, Tsinghua University, 100084 Beijing, China

**Keywords:** Nonlinear optics, Nonlinear optics

## Abstract

Antiferromagnetism has become a promising candidate for the next generation electronic devices due to its thermal stability, low energy consumption, and fast switching speed. However, the canceling of the net magnetic moment in antiferromagnetic order presents great challenge on quantitative characterization and modulation, hindering its investigation and application. In this work, utilizing the optical second harmonic generation (SHG) in a wide temperature range, the integrated differential phase contrast scanning transmission electron microscopy, and first-principles calculations, we performed a quantitative study on the evolution of non-collinear antiferromagnetic order in BiFeO_3_ films with a series of strains. We found that the antiferromagnetic coupling was significantly enhanced, featured by the increase of Néel temperature from 428 K to 646 K, and by one order of enhancement of SHG intensity contributed from the G-type antiferromagnetic order by strain manipulation from -2.4% to +0.6%. We attributed the enhancement of the antiferromagnetic coupling to the enhancement of the superexchange interaction as the Fe-O-Fe bond angle approaches 180° when the in-plane lattice constants increase, which might also result in a tendency from a non-collinear antiferromagnetic order to a collinear one. Our work not only bridges the antiferromagnetic order and the strain manipulation in epitaxial multiferroics, more importantly, also paves a way for quantitative characterization by SHG technology and the precise manipulation of antiferromagnetism.

## Abstract (web summary)

Optical second harmonic generation technology quantitatively characterized the enhancement of antiferromagnetic order and Néel temperature in epitaxially strained BiFeO_3_, providing a quantitative detection and manipulation for the antiferromagnetism.

## Graphical abstract

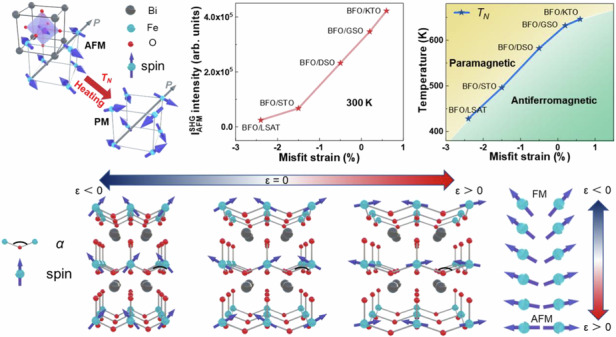

## Introduction

**Fig. 1:**
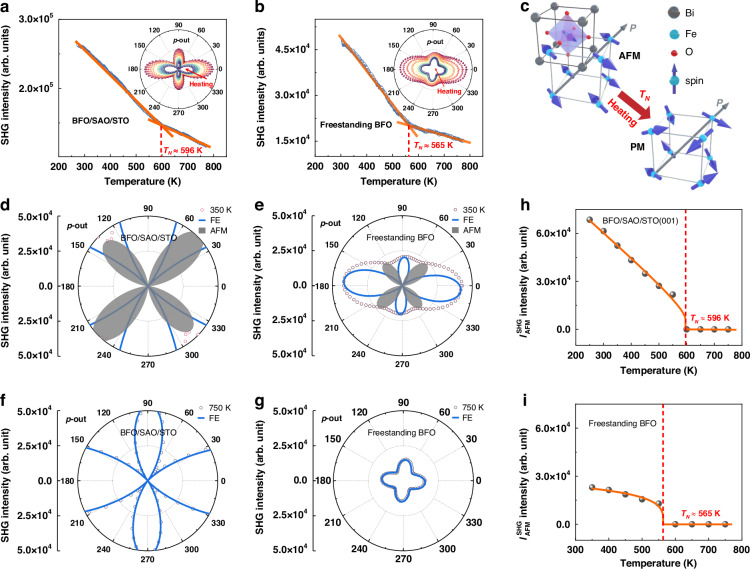
**Antiferromagnetic-paramagnetic phase transition of BFO films probed by SHG**. **a**, **b** SHG in a wide temperature range of BFO/SAO/STO films and BFO freestanding films, respectively. The insets in (**a**, **b**) are RA-SHG results of BFO/SAO/STO and freestanding BFO films at different temperatures, respectively. **c** Schematic diagram of the BFO pseudo-cubic unit cell and the antiferromagnetic-paramagnetic phase transition. **d**, **e** Theoretical fitting results of RA-SHG results of BFO/SAO/STO films and freestanding BFO films at 350 K, respectively. **f**, **g** Theoretical fitting results of RA-SHG results of BFO/SAO/STO films and freestanding BFO films at 750 K, respectively. The hollow dots represent experimental data, the bule solid lines represent the contribution from the ferroelectric order, and the gray shaded areas represent the contribution from the antiferromagnetic order. **h**, **i** Temperature-dependent SHG signals contributed from antiferromagnetic order of BFO/SAO/STO films and BFO freestanding films extracted from RA-SHG measurements, respectively

Antiferromagnetism stands out as a promising candidate for the next generation microelectronic devices owing to its robustness against external perturbations, large magnetotransport effects, and spin dynamics at the terahertz timescale^1–3^. However, due to the canceling of the net magnetic moment, it is challenging to characterize, not to mention quantitatively, and modulate the antiferromagnetism, hindering the investigation and the application of antiferromagnetism for decades^4,5^. Bismuth ferrite BiFeO_3_ (BFO), as the only room-temperature magnetoelectric multiferroic material, has attracted intense interest for the potential application in practical multifunctional devices^6,7^. At room temperature, BFO bulk has a rhombohedral phase and *R*3*c* space group in a perovskite pseudo-cubic unit cell (Fig. 1c) with lattice parameters of *a* = 3.965 Å and *α* = 89.4°^8^. Specifically, the weak magnetism of BFO originates from its non-collinear G-type antiferromagnetic structure, which is a joint effect of antiferromagnetic superexchange interaction and Dzyaloshinskii–Moriya (DM) interaction^9,10^. Intriguingly, the ferroelectricity of BFO simultaneously couples with both of these magnetic interactions, resulting in the unique magnetoelectric coupling. In spite of a number of works involving BFO and its magnetoelectric coupling, the characterization of the magnetism mainly stays in the content of the weak net magnetism rather than the non-collinear G-type antiferromagnetic order^11,12^.

Generally, optical second harmonic generation (SHG) is a nonlinear optical process that occurs when materials possess non-centrosymmetric properties and non-zero second-order nonlinear susceptibility tensors^13,14^. Analyzing the SHG signal enables effective detection and study of such symmetry breaking in materials, which is crucial for understanding their optical, electrical, and magnetic properties^15–18^. Many theoretical calculations and analyses have indicated that antiferromagnets can also generate SHG signals^19,20^. The SHG intensity ${{\boldsymbol{I}}}_{\tt{SHG}}(2\omega )$ is related to the light-induced nonlinear polarization ${\boldsymbol{P}}\left(2\omega \right)$ in the following way: ${{\boldsymbol{I}}}_{\tt{SHG}}\propto {\left|{\boldsymbol{P}}\right|}^{2}$, where ${\boldsymbol{P}}\left(2\omega \right)={\varepsilon }_{0}({\chi }^{\left({\rm{i}}\right)}+{\chi }^{\left({\rm{c}}\right)}):{\boldsymbol{E}}(\omega )\otimes {\boldsymbol{E}}(\omega )$, with ${\boldsymbol{E}}(\omega )$ denoting the incident light electric field, ${\chi }^{\left({\rm{i}}\right)}$ and ${\chi }^{\left({\rm{c}}\right)}$ denoting the time-invariant and time-noninvariant SHG tensors (Supplementary part 11), associated with the crystallographic (ferroelectric order) and G-type antiferromagnetic order, respectively^21,22^. Subsequently, several experimental works utilize the SHG method to study antiferromagnets^23,24^. Therefore, SHG methods are a powerful tool for characterizing antiferromagnets. However, there are few studies on how to effectively tune the strength of antiferromagnetism and the antiferromagnetic phase transition with epitaxial strains characterized by SHG. In BFO, the G-type antiferromagnetic order also contributes to the spatial inversion asymmetry via spin-orbital coupling, which allows us to quantitatively characterize, through the SHG measurements, how strain manipulates the antiferromagnetic order^21,22^.

In 2010, I. C. Infante et al. reported that the magnetic Nèel temperature of epitaxial BFO films showed almost no change with strain^25^. The significant differences in results are primarily due to the large temperature step of ~50 K and less data collected in the neutron diffraction measurements in the previous work. Because the Néel temperatures were determined by the intensity of neutron diffraction as a function of temperature in their work, it is difficult to precisely capture the antiferromagnetic transition. Besides, in 2011, I. C. Infante et al. reported that the Néel temperature of BFO/LaAlO_3_(001) films under high compressive stress was 360 ± 20 K^26^, indicating clear influence of the Néel temperature by the strain. However, when the variation in strain is small, more sensitive detection method with finer temperature resolution are required. The optical SHG method has significant advantages in characterizing antiferromagnetic phase transitions due to its lack of requirements for sample size and its high sensitivity to symmetry change. The conveniency of SHG measurements allows us to characterize the antiferromagnetism with a wider temperature range and a much higher temperature resolution.

In this work, we performed a systematic study on the evolution of non-collinear antiferromagnetic order in BFO films with a series of strains by the measurements of SHG in a wide temperature range, rotational-anisotropy SHG (RA-SHG), and the integrated differential phase contrast scanning transmission electron microscopy (iDPC-STEM) characterization. We found that the Néel temperature (${T}_{{\rm{N}}}$), at which the thermal fluctuation destroys the antiferromagnetic order, increased from 428 K to 646 K with the manipulation of in-plane biaxial strain from -2.4% to +0.6%. Meanwhile, the SHG intensity contributed from the G-type antiferromagnetic order (${I}_{{\rm{AFM}}}^{{\rm{SHG}}}$) increased by one order, also exhibited a monotonically increasing correlation with the in-plane lattice constants, quantitatively indicating the significant enhancement of antiferromagnetic coupling. We attributed the enhancement of antiferromagnetic coupling to the enhancement of the superexchange interaction^27,28^, as the Fe-O-Fe bond angle approaches 180° when the in-plane lattice constants increase, which was confirmed by the iDPC-STEM and by our first-principles calculation. Besides, we think the increase of Fe-O-Fe bond angle might also result in a tendency from a non-collinear antiferromagnetic order to a collinear one^29,30^, leading to the further enhancement of antiferromagnetic coupling and the increase of ${T}_{{\rm{N}}}$^27,31^. Our work demonstrates the evolution of antiferromagnetic order with the strain manipulation in epitaxial multiferroic films, more importantly, also paves a way for characterizing the antiferromagnetism with Zero net magnetic moment quantitatively by optical SHG technology.

## Results

### Antiferromagnetic-paramagnetic phase transition probed in BFO films

The G-type antiferromagnetic order in BFO films gradually weakens as temperature increases and is destroyed eventually at ${T}_{{\rm{N}}}$, leading to a magnetic phase transition to paramagnetic one^22^. To determine the antiferromagnetic-paramagnetic phase transition, we performed SHG measurements (all the SHG measurements in this work were performed in the *p*-in/*p*-out polarization configuration, where *p*- is parallel to the plane of incidence) in a wide temperature range for BFO/Sr_4_Al_2_O_7_ (SAO)/SrTiO_3_ (STO) films and freestanding BFO films. Both SAO and BFO films were deposited sequentially on STO (001) substrates by pulsed laser deposition. The thicknesses of the SAO layer and the BFO layer are confirmed to be ~15 nm and 50 nm by X-ray reflectivity measurement, respectively. The thermal fluctuation destroys the G-type antiferromagnetic order in BFO bulk at ${T}_{{\rm{N}}} \sim 640\ {\rm{K}}$, while the ferroelectricity persists at ${T}_{{\rm{C}}} \sim 1100\ {\rm{K}}$^25^. Consequently, as shown in Fig. 1a and Fig. 1b, the intensity of SHG as a function of temperature shows a singular point at 596 K and 565 K for BFO/SAO/STO films and freestanding BFO films, respectively, indicating an antiferromagnetic-paramagnetic phase transition at these temperatures (Fig. 1c). To confirm and further understand this phase transition, we performed rotational-anisotropy SHG (RA-SHG) measurements for BFO/SAO/STO films and freestanding BFO ones at a series of temperatures from 200 K to 750 K with an interval of 50 K as shown in the insets of Fig. 1a and Fig. 1b, respectively. The RA-SHG patterns of BFO/SAO/STO films and freestanding BFO films exhibit an asymmetry at low temperature and become symmetric as the temperature increases. For instance, the RA-SHG fitting results at 350 K indicate the coexistence of ferroelectric order and G-type antiferromagnetic order in both BFO/SAO/STO films and BFO freestanding films as shown in Fig. 1d and Fig. 1e, respectively (detailed RA-SHG fitting results are shown in Figs. S1 and S2). On the contrary, the RA-SHG fitting results at 750 K contain only the contribution from the ferroelectric order in both BFO/SAO/STO films and freestanding BFO films as shown in Fig. 1f and Fig. 1g, respectively.

The contribution from the ferroelectric order and that from the G-type antiferromagnetic order to the SHG signals were determined separately. As shown in Fig. 1h, i, the SHG intensity of the contribution from the antiferromagnetic order decreases with the increase of temperature, and eventually falls to zero. We fitted these data with a well-known phenomenological function^16,32^ ${I}_{{\rm{S}}{\rm{H}}{\rm{G}}}^{2\omega }\propto {[a+b{({T}_{{\rm{N}}}-T)}^{\beta }]}^{2}$, from which ${T}_{{\rm{N}}}$ were determined and confirmed to be 596 K and 565 K for BFO/SAO/STO films and BFO freestanding films, respectively. Besides, it is worth noting that ${I}_{{\rm{AFM}}}^{{\rm{SHG}}}$ of the BFO/SAO/STO films is ~2.3 times larger than that of the freestanding BFO ones at 350 K, indicating stronger antiferromagnetic order in BFO/SAO/STO films. The higher ${T}_{{\rm{N}}}$ and larger ${I}_{{\rm{AFM}}}^{{\rm{SHG}}}$ before releasing the epitaxial strain suggests the potential of strain manipulation for the antiferromagnetism.

### Growth and structure characterization of epitaxial BFO films

To investigate the strain manipulation of the antiferromagnetic order, and by considering the intolerance of the freestanding BFO on polydimethylsiloxane (PDMS) with strain at high temperature, we epitaxially grew BFO films on a series of substrates including (LaAlO_3_)_0.3_-(Sr_2_AlTaO_6_)_0.7_ (LSAT), STO, DyScO_3_ (DSO), GdScO_3_ (GSO), and KTaO_3_ (KTO) via pulsed laser deposition. The in-plane lattice constants and the corresponding misfit strain $\varepsilon =({a}_{{\rm{sub}}}-{a}_{{\rm{BFO}}})/{a}_{{\rm{BFO}}}$ of these substrates are 3.868 Å (-2.4%), 3.905 Å (-1.5%), 3.944 Å (-0.5%), 3.973 Å (+0.2%), and 3.989 Å (+0.6%), respectively, where ${a}_{\tt{sub}}$ is the in-plane lattice constants of the substrate and ${a}_{{\rm{BFO}}}=3.965{\text{\AA}}$ is the pseudocubic lattice constants of bulk BFO. To be noted, in order to reduce the effect of BFO films thickness on optical measurements and avoid structural relaxation, the thickness of the BFO films grown on all substrates was set to be ~30 nm for all samples (see Fig. S3 for X-ray reflectivity results).

**Fig. 2:**
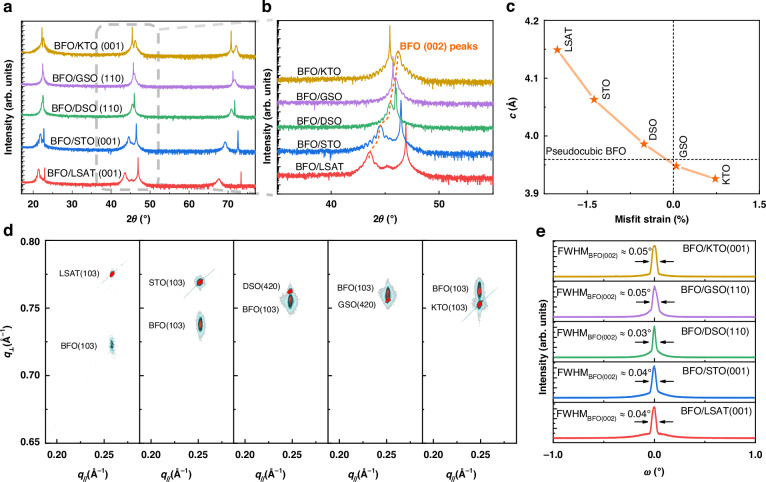
**Structural characterization of BFO films grown on different substrates. a** Wide-range XRD *θ*-2*θ* scans of BFO films epitaxially grown on LSAT, STO, DSO, GSO, and KTO substrates, respectively. **b** (002) diffraction peaks of BFO films grown on different substrates, where obvious shifts are shown by the orange dashed lines. **c** Out-of-plane lattice constant *c* as a function of the in-plane strain of BFO films grown on different substrates. **d** RSMs around the (103) diffraction peaks of BFO films grown on different substrates, respectively. **e** Rocking curves around the (002) diffraction peaks of BFO films grown on different substrates

The high epitaxial quality of the BFO films was exhibited by the results of X-ray diffraction (XRD) as shown in Fig. 2a. With the increase of in-plane lattice constants, the out-of-plane constants *c* of BFO decrease (from 4.149 Å–3.926 Å), leading to the shift of (002) BFO peaks towards higher $2\theta$ as shown in Fig. 2b, c. We also performed the reciprocal space mappings (RSMs), where the peaks of the substrates and the BFO locate in identical ${q}_{//}$ (in-plane reciprocal-space) (Fig. 2d), suggesting the identical in-plane lattice constants and well-applied strains. Besides, the full width at half maximum (FWHM) values for the 001 peaks of BFO films grown on various substrates were within 0.03-0.05°, which was comparable to that (~0.02°) of the single-crystalline substrates, indicating excellent crystallinity of the BFO films (Fig. 2e).

### Quantitative characterization of G-type antiferromagnetism by SHG

**Fig. 3:**
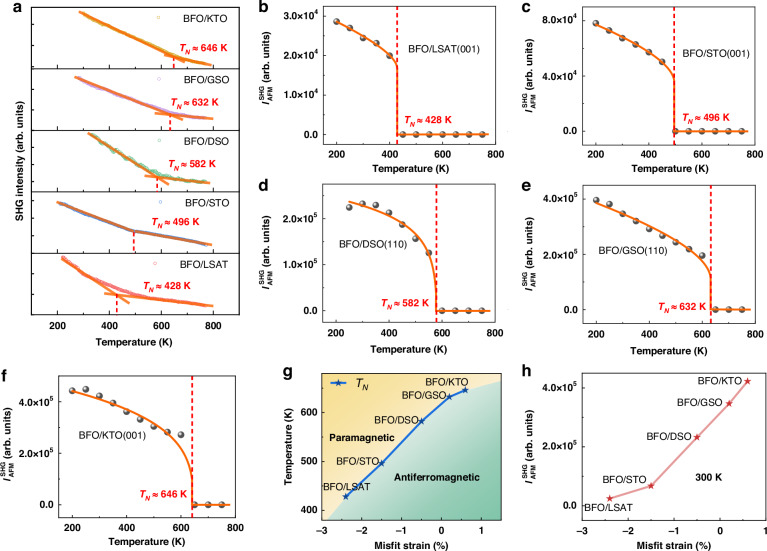
**Temperature-dependent optical SHG measurements of BFO films grown on different substrates. a** SHG results in a wide temperature range of BFO films grown on LSAT, STO, DSO, GSO, and KTO substrates, respectively. **b**–**f** Temperature-dependent SHG signals contributed from antiferromagnetic order of BFO films grown on LSAT, STO, DSO, GSO, and KTO substrates, respectively. The orange solid lines are fitted results. **g** ${T}_{{\rm{N}}}$ as a function of misfit strain for BFO films grown on different substrates. **h** SHG intensity contributed from the antiferromagnetic order of BFO films grown on different substrates as a function of misfit strain

To be noted, since the laser spot size in our experiments is ~100 μm, which is much larger than those of the ferroelectric and antiferromagnetic domains^33,34^, what we observe here is an average effect contributed by uniformly-existed possible domains together. To investigate the evolution of G-type antiferromagnetic order in these epitaxial BFO films, we employed SHG in a wide temperature range from 200 K–750 K continuously (Fig. 3a) and RA-SHG measurements from 200 K–750 K with an interval of 50 K (Fig. 3b–f and Fig. S4–S8). In Fig. 3a, ${T}_{{\rm{N}}}$ of these BFO films were firstly determined by extracting the turning point of the temperature-dependent SHG intensity curve, with high precision. Then, the SHG intensity contributions from the G-type antiferromagnetic order, which were extracted from the RA-SHG measurements as a function of temperature (with an interval of 50 K), were also fitted using a well-known phenomenological function^16,32^ ${I}_{{\rm{S}}{\rm{H}}{\rm{G}}}^{2\omega }\propto {[a+b{({T}_{{\rm{N}}}-T)}^{\beta }]}^{2}$ to obtain ${T}_{{\rm{N}}}$. By combining the analyzation of those SHG measurements, ${T}_{{\rm{N}}}$ of the BFO films with strain of $\varepsilon =-2.4 \%$,$-1.5 \%$, $-0.5 \%$, $+0.2 \%$, and $+0.6 \%$ were obtained to be 428 K, 496 K, 582 K, 632 K, and 646 K, respectively, as shown in Fig. 3b–f. Namely, ${T}_{{\rm{N}}}$ increases with the in-plane lattice constants (Fig. 3g). Furthermore, ${I}_{{\rm{SHG}}}^{{\rm{AFM}}}$ also exhibits a monotonically increasing correlation with in-plane lattice constants as shown in Fig. 3h, and its magnitude is one order larger for $\varepsilon =+0.6 \%$ (on KTO) than that for $\varepsilon =-2.4 \%$ (on LSAT), quantitatively demonstrating the significant enhancement of the antiferromagnetism. Thus, very importantly, the antiferromagnetism with Zero net magnetic moment can now be quantitatively described by SHG measurements.

### Mechanism of strain manipulation of the antiferromagnetic order

**Fig. 4:**
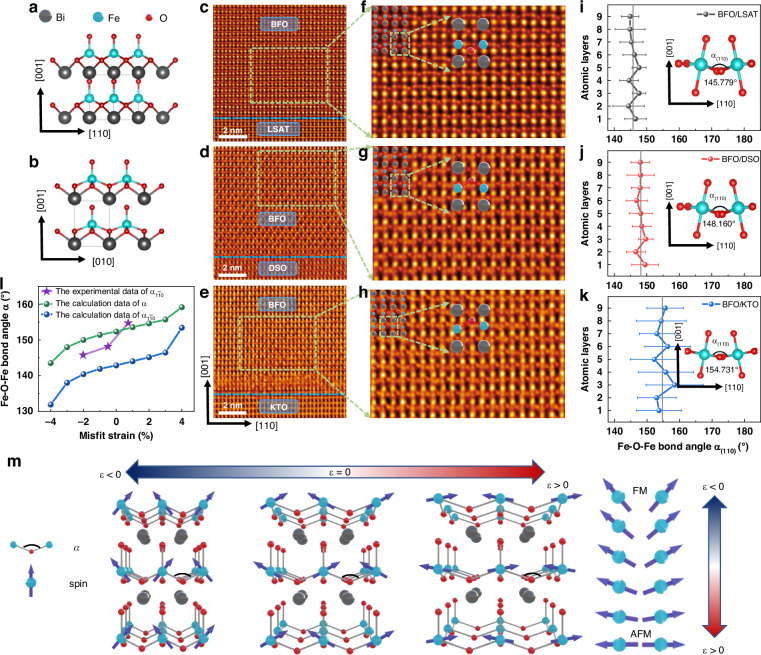
**Characterization of Fe-O-Fe bond angle in strain manipulated BFO films. a**, **b** Atomic structure schematic of tetragonal BFO projected along the $\left[1\overline{1}0\right]$ and [100] directions, respectively. **c**–**e** Cross-sectional iDPC-STEM images of BFO/LSAT, BFO/DSO, and BFO/KTO films, respectively. **f**–**h** Magnified views of the framed areas in (**c**–**e**). **i**–**k** Statistical average of Fe-O-Fe bond angle projected on the $(1\bar{1}0)$ plane ${\alpha }_{1\overline{1}0}$ for BFO/LSAT, BFO/DSO, and BFO/KTO films, respectively. The error bar represents the standard deviation of measured atomic layers. **l** ${\alpha }_{1\overline{1}0}$ obtained from iDPC-STEM (purple star), Fe-O-Fe bond angle$\alpha$ (green dots) and ${\alpha }_{1\overline{1}0}$ (blue dots) obtained from first-principles calculations, as a function of misfit strain. **m** Schematic diagram of the arrangement of the nearest neighbor Fe^3+^ spins in BFO films with different strain

According to the Goodenough-Kanamori rule^35^, Fe^3+^ ions in BFO, where all five 3 *d* orbitals are half-filled, exhibit a strong antiferromagnetic superexchange interaction with the near-180° Fe-O-Fe bond angle (labeled as $\alpha$). To investigate how the strain manipulates the antiferromagnetic properties, $\alpha$ as a function of strain should be addressed. We performed atomic-resolved iDPC-STEM imaging for BFO/LSAT, BFO/DSO, and BFO/KTO films. The projection direction was chosen to be along $[1\bar{1}0]$ (Fig. 4a) rather than $[100]$ (Fig. 4b) to measure the FeO_6_ octahedra tilt for better recognition of $\alpha$. As shown in Fig. 4c–e, all the iDPC-STEM images of BFO/LSAT, BFO/DSO, and BFO/KTO films showed atomically sharp interfaces between the BFO layers and the substrates. The framed sections were magnified as shown Fig. 4f–h, where the atoms were marked by dots with different colors. By determining the positions of Fe and O atoms, the statistical average of $\alpha$ projected on the $(1\bar{1}0)$ plane, ${\alpha }_{1\overline{1}0}$, were calculated for each layer as shown in Fig. 4i, k. Specifically, ${\alpha }_{1\overline{1}0}$ for the BFO/LSAT, BFO/DSO, and BFO/KTO films are 145.779°, 148.160°, and 154.731°, respectively. Namely, ${\alpha }_{1\overline{1}0}$ increases as the in-plane lattice constants increase. Furthermore, we performed first-principles calculations to obtain the structure of BFO with various strains ranging from -4% to 4% (Figs. S9 and Fig. S10). We extracted $\alpha$ and ${\alpha }_{1\overline{1}0}$ as a function of strain (Fig. 4l), which suggests that both $\alpha$ and ${\alpha }_{1\overline{1}0}$ increase with the increase of in-plane lattice constants, consisting with our experimental results.

We think the antiferromagnetic order in BFO was manipulated by strain in two aspects. On one hand, since the antiferromagnetic superexchange interaction favors $\alpha$ being 180°, the increase of in-plane lattice constants enhances the antiferromagnetic superexchange interaction. On the other hand, the larger $\alpha$ might resulting in a weaker DM interaction. Since the non-collinear G-type antiferromagnetic structure in BFO is a joint effect of antiferromagnetic superexchange interaction and DM interaction, the weaker DM interaction and the stronger antiferromagnetic superexchange interaction enhance the non-collinearity of antiferromagnetic order (less inclined neighboring Fe^3+^ spins) as schematically shown in Fig. 4m. To sum up, the G-type antiferromagnetic order can be greatly enhanced by strain manipulation, characterized by the increase of ${T}_{{\rm{N}}}$ and ${I}_{{\rm{AFM}}}^{{\rm{SHG}}}$.

## Conclusion

By investigating the evolution of non-collinear antiferromagnetic order in BFO films with a series of strains, we found that ${T}_{{\rm{N}}}$ could be manipulated from 428 K to 646 K by the strain from $-2.4 \%$ to $+0.6 \%$. Meanwhile the intensity of SHG contributed from the G-type antiferromagnetic order ${I}_{{\rm{SHG}}}^{{\rm{AFM}}}$ increased with the magnitude of one order, and also exhibited a monotonically increasing correlation with in-plane lattice constants. The combination of ${T}_{{\rm{N}}}$ and ${I}_{{\rm{SHG}}}^{{\rm{AFM}}}$ quantitatively demonstrated an enhancement of the G-type antiferromagnetic order. By performing iDPC-STEM measurements for BFO/LSAT, BFO/DSO, and BFO/KTO films, we found that the Fe-O-Fe bond angle projected on the $(1\overline{1}0)$ plane ${\alpha }_{1\overline{1}0}$ increased from 145.779°–154.731° as the in-plane lattice constants increase from 3.868 Å–3.989 Å. Our first-principles calculation for BFO with in-plane strains ranging from -4% to 4% also confirmed this positive correlation. We attributed the enhancement of antiferromagnetic coupling to the enhancement of superexchange interaction as the Fe-O-Fe bond angle $\alpha$ increases and approaches 180° with the increase of in-plane lattice constants. We also think that the increase of $\alpha$ might results in a tendency from a non-collinear antiferromagnetic order to a collinear one. Our work demonstrates the manipulation of antiferromagnetic order by strain in epitaxial multiferroics, and more importantly, paves a way for quantitatively measuring the antiferromagnetism with Zero magnetic moment using optical SHG technology.

## Methods

### Thin films synthesis and structural characterizations

Both SAO and BFO films were deposited sequentially on STO (001) substrates by pulsed laser deposition using a XeCl excimer laser with a wavelength of 248 nm. The water-sacrificial SAO layer was grown at 780 °C under an oxygen pressure of 2.0 Pa with a substrate-target distance of 7.5 cm, the energy density was about 2.2 J/cm^2^, and the repetition rate was 2 Hz. For all samples, the BFO layers with a series of strains were deposited under identical experimental condition, i.e. temperature of substrate ${T}_{{\rm{sub}}}=700\ {\circ \atop} {\rm{C}}$, oxygen pressure ${P}_{{{\rm{O}}}_{2}}=20.0\ {\rm{Pa}}$, and laser energy ${E}_{{\rm{laser}}}=1.6\ {\rm{J}}/{\rm{c}}{{\rm{m}}}^{2}$. After growth, the films were post-annealed under the growth condition of the BFO layer for 30 min to maintain their stoichiometry and then slowly cooled to room temperature. The BFO/SAO/STO epitaxial films were immersed directly in deionized water for about 30 min. Following complete dissolution of the sacrificial layer SAO, the BFO films were released from the STO (001) substrates and transferred onto target substrates (e.g., flexible substrate PDMS) placed on a 95 °C hot plate. After the water evaporation, the freestanding BFO films remained on the target substrate.

XRD, RSM, *ω*-scans, and X-ray reflectivity measurements of the BFO/SAO/STO films, freestanding BFO films, and BFO films epitaxially grown on different substrates were performed using a Panalytical X’Pert3 MRD diffractometer with Cu-Kα_1_ (1.54056 Å) radiation equipped with a 3D pixel detector.

### Electron microscopy

The iDPC-STEM imaging was conducted using Cs-corrected (S)TEM (FEI Titan Cubed Themis G2 300), operated at 300 kV. The collection angle for iDPC-STEM imaging is 21 mrad. Integrated differential phase contrast imaging technology used differential phase contrast (DPC) imaging of the four-quadrant detector in STEM^36^. By physically analyzing the electrons collected in the bright field area, the *x* and *y* components of the DPC image are calculated, and the final image is obtained after two-dimensional integration^37^.

### Optical second harmonic generation measurements

The wide-temperature-range (RA-)SHG measurements on BFO films were all conducted in a typical 45° reflection geometry (Fig. S11). The incident femtosecond laser beam, generated by the Maitai SP Ti:Sapphire oscillator produced by Spectra Physics, was tuned with the incident light power maintained at 50 mW, the center wavelength of 800 nm, the pulse width of 120 fs, and the frequency of 82 MHz. The polarization direction $\varphi$ of the incident light field was adjusted by rotating the $\lambda /2$ waveplate with a rotary motor. The second harmonic signal was collected by a photomultiplier tube and transmitted to a photon counter. The reflected light was fixed as *p* or *s* (*p*-, parallel and *s*-, perpendicular to the plane of incidence) polarization, and the rotated anisotropy patterns under different reflection polarization configurations were obtained by rotating the incident light polarization angle $\varphi$.

The temperature-variable stage used in the SHG measurement process is a Heating and Cooling Stage, i.e., model HFS600EPB4 produced by LINKAM, UK, with a broad temperature range of 77–873 K.

### First-principles calculations

The density functional theory calculations were performed using generalized-gradient-approximation with the PBEsol functional^38^ which is optimized for surfaces and solids as implemented in the SIESTA package^39,40^. We use double-zeta- polarized (DZP) numerical atomic orbitals as the basis set for each atom. The Hubbard U correction method^41^ were used to improve the description of the onsite interaction of the Fe 3 *d* orbitals represented by pseudo-atomic-orbitals^42^, with a typically used $U({\rm{Fe}})=4\ {\rm{eV}}$ and $J({\rm{Fe}})=0.4\ {\rm{eV}}$^43,44^. The scalar-relativistic norm-conserving pseudopotentials from the Pseudo-Dojo dataset^45^ are used. We use 2 × 2 × 2 pseudo-cubic supercells of BFO with 40 atoms per cell. To mimic the epitaxial strain effect, the structures are relaxed with the in-plane lattice parameters constrained to various strain values. The reference of the strain is the pseudo-cubic lattice parameter of BFO as from DFT relaxation result. The structures are relaxed with until the forces are <0.001 eV/Å.

## Supplementary information

Supplementary Information

## Data Availability

The data that support the findings of this study are available on the proper request from the first author (S.X.) and the corresponding authors (K.J.).
