# Supplementary material for: Characterizing G-type antiferromagnetism quantitatively with optical second harmonic generation
Source: Light Sci Appl. 2025 Apr 22;14:169. doi: 10.1038/s41377-025-01849-3 (PMC12015458; doi:10.1038/s41377-025-01849-3)
Supplement: Supplementary file 1 — Supplementary Information [file 41377_2025_1849_MOESM1_ESM.pdf]

SUPPLEMENTARY INFORMATION for

Characterizing G-type antiferromagnetism quantitatively with optical second harmonic generation

Shuai Xu[1, †], Cheng Ma[1,2, †], Kui-juan Jin[1,2,*], Qinghua Zhang[1,2], Sisi Huang[1,2], Yiru Wang[1,2], Xu He[3], Jiesu Wang[4], Donggang Xie[1,2], Qiulin Zhang[1,2], Er-Jia Guo[1,2], Chen Ge[1,2], Can Wang[1,2], Xiulai Xu[5], Lin Gu[6], Meng He[1], Guozhen Yang[1,2]

[1] Beijing National Laboratory for Condensed Matter Physics, Institute of Physics, Chinese Academy of Sciences, Beijing 100190, China

[2] University of Chinese Academy of Sciences, Beijing 100049, China

[3]Theoretical Materials Physics, Q-MAT, Université de Liège, Liège B-4000, Belgium

[4]Beijing Academy of Quantum Information Sciences, Beijing 100193, China

[5]State Key Laboratory for Mesoscopic Physics and Frontiers Science Center for Nano-optoelectronics, School of Physics, Peking University, Beijing 100871, China

[6]Beijing National Center for Electron Microscopy and Laboratory of Advanced Materials, Department of Materials Science and Engineering, Tsinghua University, 100084, Beijing, China

† These authors contributed equally: Shuai Xu, Cheng Ma

*Author to whom correspondence should be addressed: kjjin@iphy.ac.cn

CONTENTS

1. RA-SHG measurements of BFO/SAO/STO heterostructures at different temperatures
2. RA-SHG measurements of freestanding BFO films at different temperatures
3. X-ray reflectivity measurements of BFO films grown on different substrates
4. RA-SHG measurements of BFO/LSAT films at different temperatures
5. RA-SHG measurements of BFO/STO films at different temperatures
6. RA-SHG measurements of BFO/DSO films at different temperatures
7. RA-SHG measurements of BFO/GSO films at different temperatures
8. RA-SHG measurements of BFO/KTO films at different temperatures
9. First-principles calculation results of the crystal structures of BFO with different strains
10. $(1\bar{1}0)$ plane-view of the BFO crystal structure with different strains
11. Functional forms of electric dipole SHG for crystallographic 4*mm* and magnetic point-group *m*

1. RA-SHG measurements of BFO/SAO/STO films at different temperatures

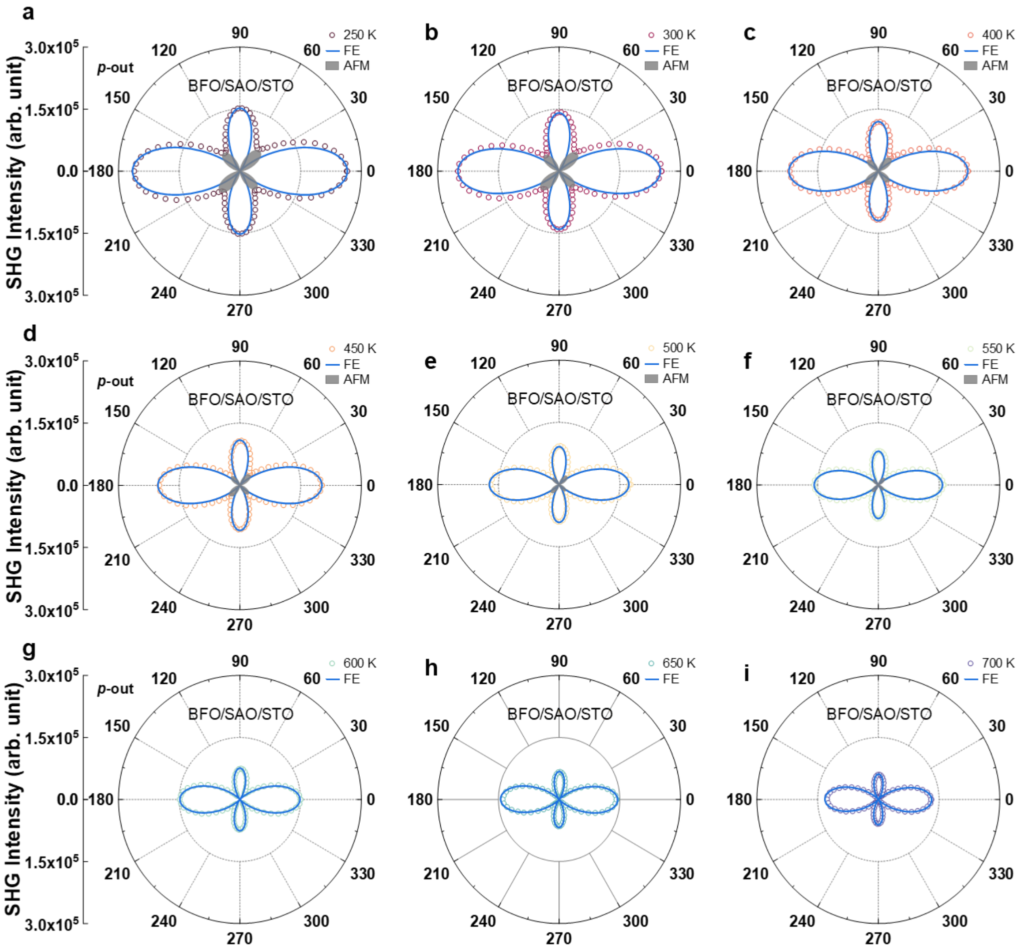

Supplementary Figure 1 | a-f RA-SHG results and analyses of BFO/SAO/STO films below T_N (about 596 K for BFO/SAO/STO films), where both the antiferromagnetic order and the ferroelectric order contribute to the SHG signal, leading to the asymmetry of the RA-SHG patterns. **g-i** RA-SHG results and analyses of BFO/SAO/STO films above T_N, where contribution from the antiferromagnetic order disappears and only the ferroelectric order contributes to the SHG signal. The hollow dots are the experimental data, the blue lines represent the contribution from the ferroelectric order, and the gray shadows represent the contribution from the antiferromagnetic order.

2. RA-SHG measurements of freestanding BFO films at different temperatures

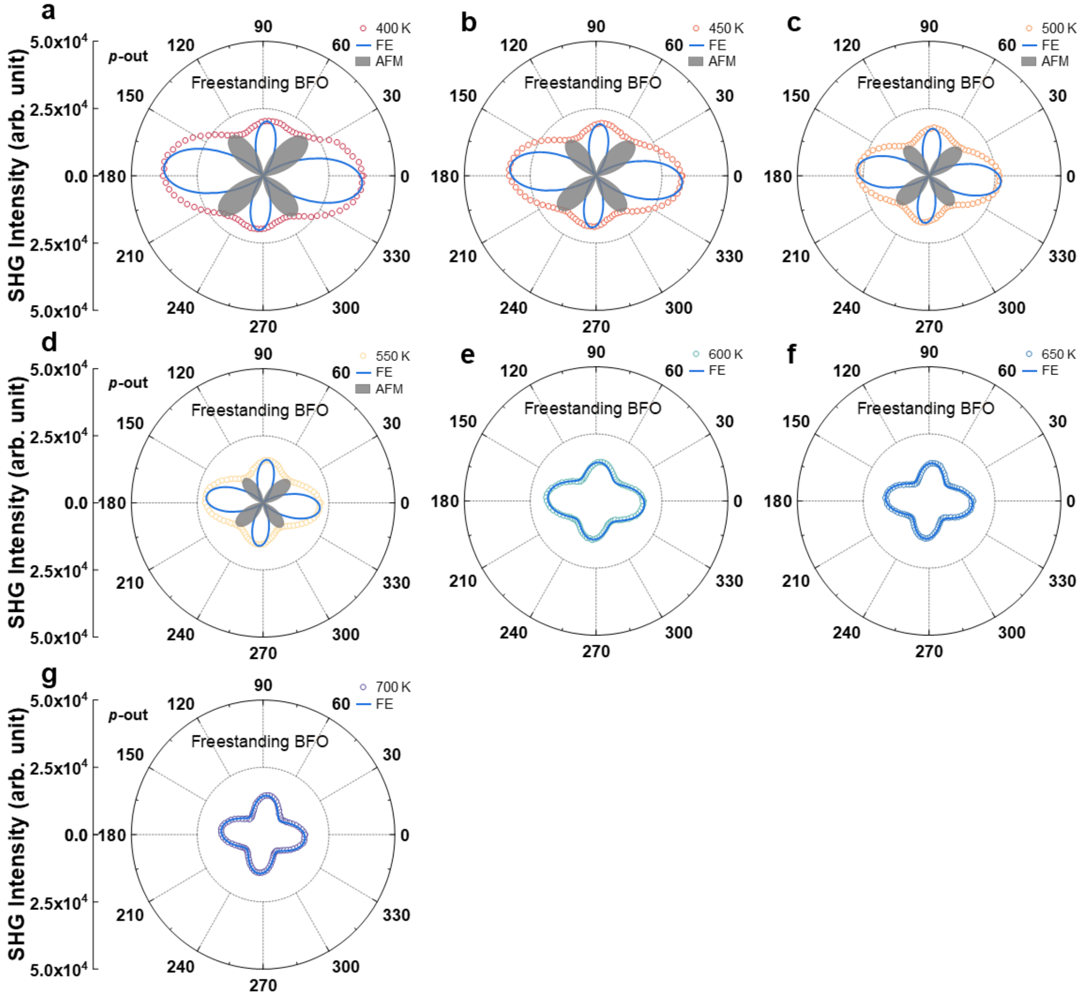

Supplementary Figure 2 | a-d RA-SHG results and analyses of freestanding BFO films below T_N (about 565 K for freestanding BFO films), where both the antiferromagnetic order and the ferroelectric order contribute to the SHG signal, leading to the asymmetry of the RA-SHG patterns. **e-g** RA-SHG results and analyses of freestanding BFO films above T_N, where contribution from the antiferromagnetic order disappears and only the ferroelectric order contributes to the SHG signal. The hollow dots are the experimental data, the blue lines represent the contribution from the ferroelectric order, and the gray shadows represent the contribution from the antiferromagnetic order.

3. X-ray reflectivity measurements of BFO films grown on different substrates

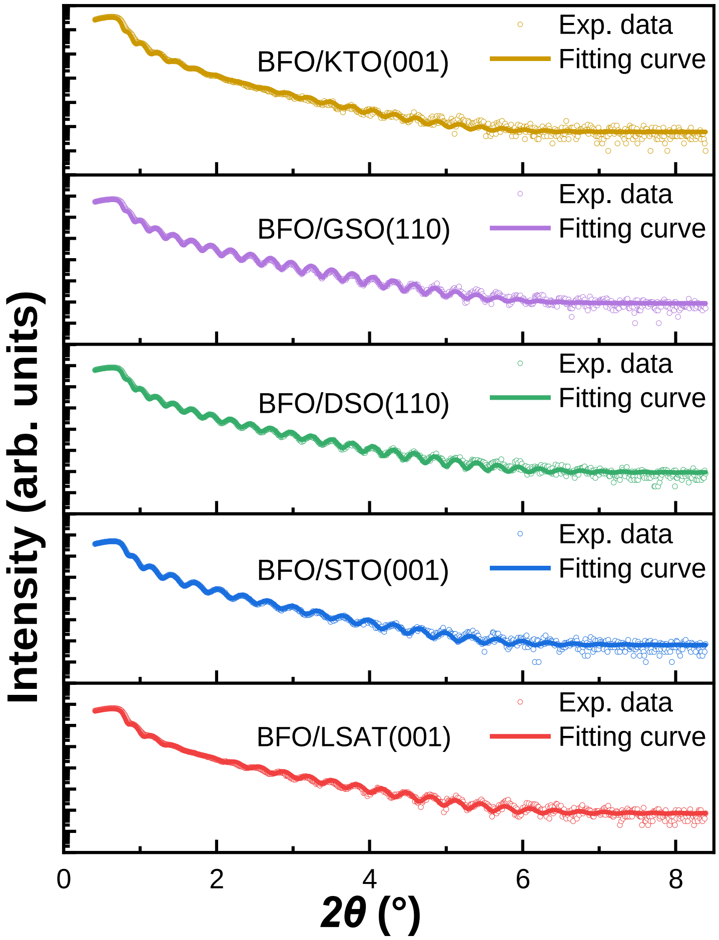

Supplementary Figure 3 | X-ray reflectivity measurements of BFO/LSAT, BFO/STO, BFO/DSO, BFO/GSO, and BFO/KTO films, respectively. The lines are the fitting curves to the experimental data (hollow dots). The thickness of all epitaxial BFO films on different substrates is approximately 30 nm.

4. RA-SHG measurements of BFO/LSAT films at different temperatures

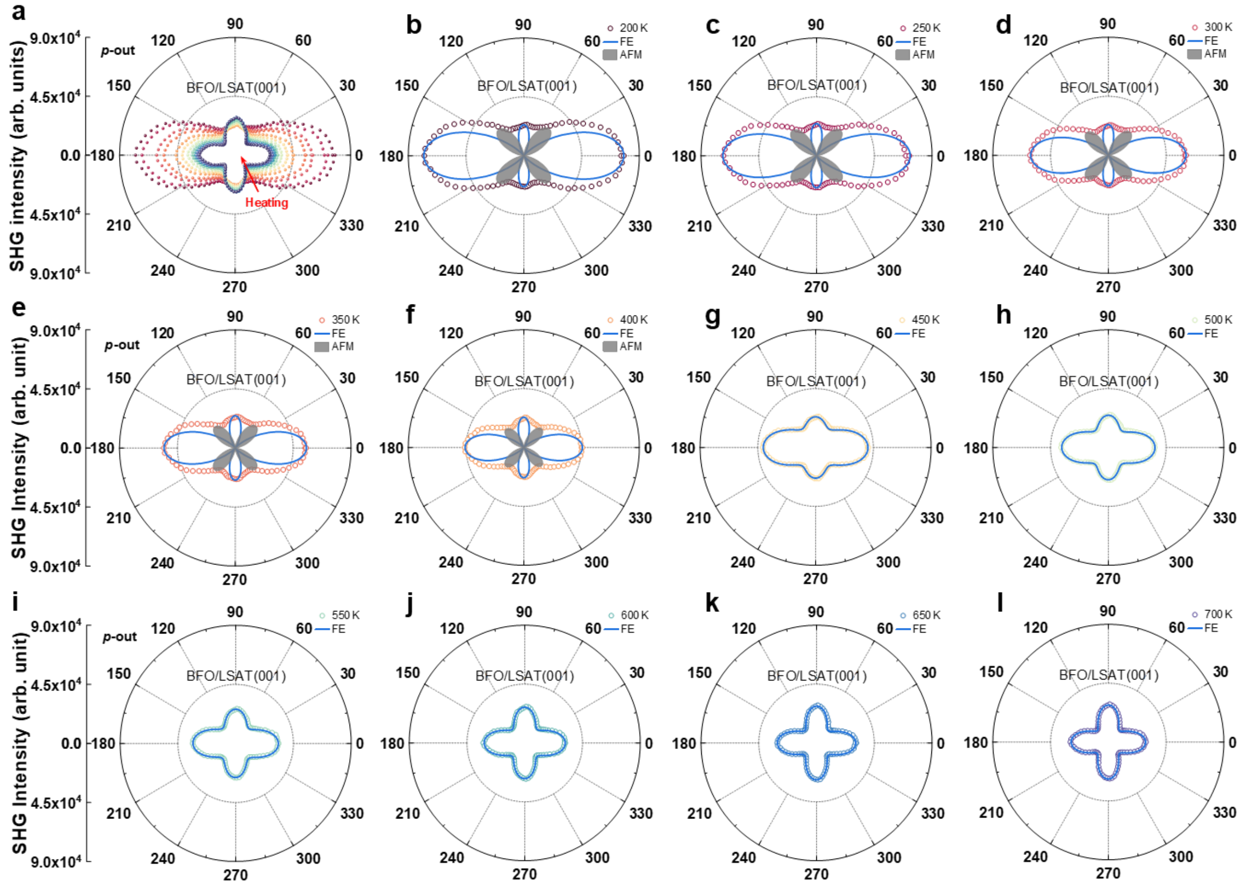

Supplementary Figure 4 | a RA-SHG results of BFO/LSAT films at different temperatures. **b-f** RA-SHG results and analyses of BFO/LSAT films below T_N (about 428 K for BFO/LSAT films), where both the antiferromagnetic order and the ferroelectric order contribute to the SHG signal, leading to the asymmetry of the RA-SHG patterns. **g-l** RA-SHG results and analyses of BFO/LSAT films above T_N, where contribution from the antiferromagnetic order disappears and only the ferroelectric order contributes to the SHG signal. The hollow dots are the experimental data, the blue lines represent the contribution from the ferroelectric order, and the gray shadows represent the contribution from the antiferromagnetic order.

5. RA-SHG measurements of BFO/STO films at different temperatures

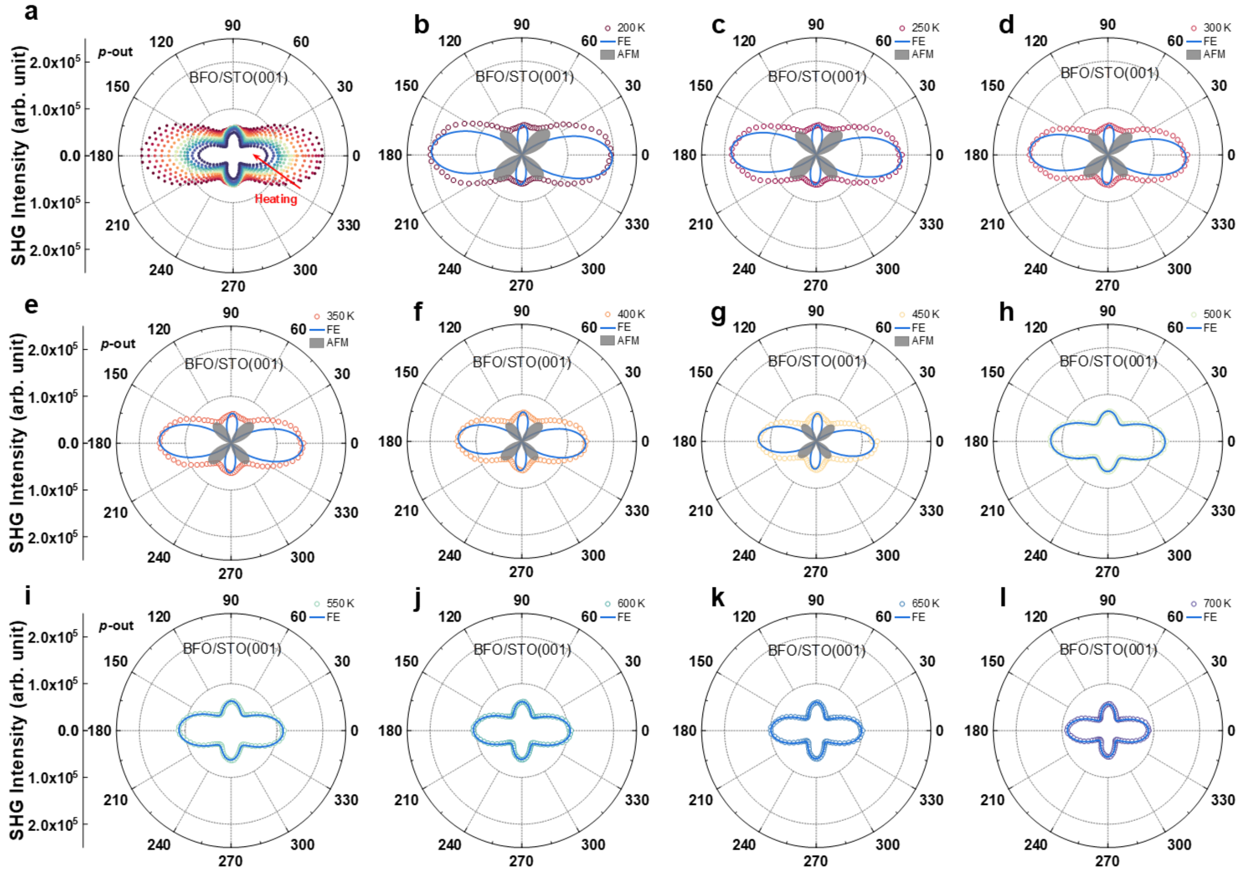

Supplementary Figure 5 | a RA-SHG results of BFO/STO films at different temperatures. **b-g** RA-SHG results and analyses of BFO/STO films below T_N (about 496 K for BFO/STO films), where both the antiferromagnetic order and the ferroelectric order contribute to the SHG signal, leading to the asymmetry of the RA-SHG patterns. **h-l** RA-SHG results and analyses of BFO/STO films above T_N, where contribution from the antiferromagnetic order disappears and only the ferroelectric order contributes to the SHG signal. The hollow dots are the experimental data, the blue lines represent the contribution from the ferroelectric order, and the gray shadows represent the contribution from the antiferromagnetic order.

6. RA-SHG measurements of BFO/DSO films at different temperatures

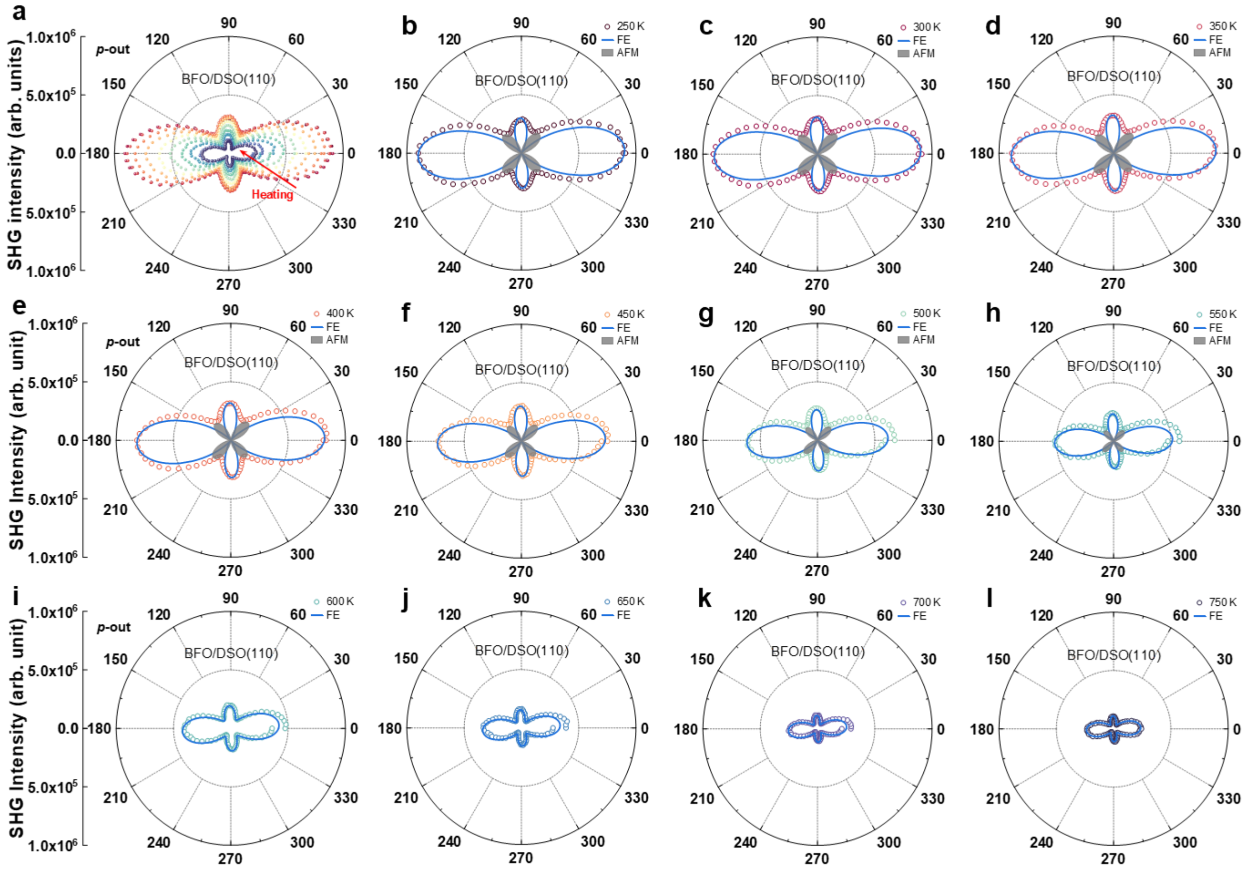

Supplementary Figure 6 | a RA-SHG results of BFO/DSO films at different temperatures. **b-h** RA-SHG results and analyses of BFO/DSO films below T_N (about 582 K for BFO/DSO films), where both the antiferromagnetic order and the ferroelectric order contribute to the SHG signal, leading to the asymmetry of the RA-SHG patterns. **i-l** RA-SHG results and analyses of BFO/DSO films above T_N, where contribution from the antiferromagnetic order disappears and only the ferroelectric order contributes to the SHG signal. The hollow dots are the experimental data, the blue lines represent the contribution from the ferroelectric order, and the gray shadows represent the contribution from the antiferromagnetic order.

7. RA-SHG measurements of BFO/GSO films at different temperatures

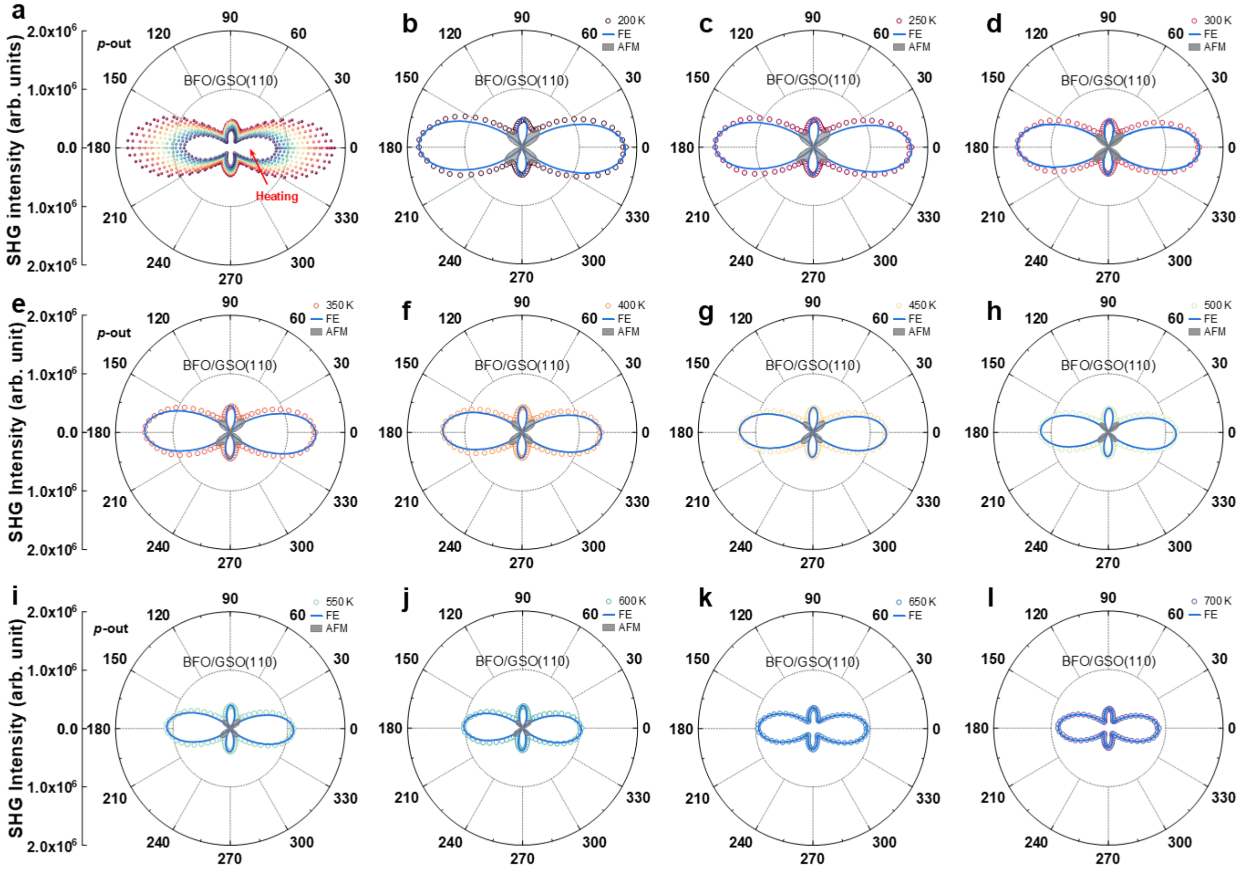

Supplementary Figure 7 | a RA-SHG results of BFO/GSO films at different temperatures. **b-j** RA-SHG results and analyses of BFO/GSO films below T_N (about 632 K for BFO/GSO films), where both the antiferromagnetic order and the ferroelectric order contribute to the SHG signal, leading to the asymmetry of the RA-SHG patterns. **k** and **l** RA-SHG results and analyses of BFO/GSO films above T_N, where contribution from the antiferromagnetic order disappears and only the ferroelectric order contributes to the SHG signal. The hollow dots are the experimental data, the blue lines represent the contribution from the ferroelectric order, and the gray shadows represent the contribution from the antiferromagnetic order.

8. RA-SHG measurements of BFO/KTO films at different temperatures

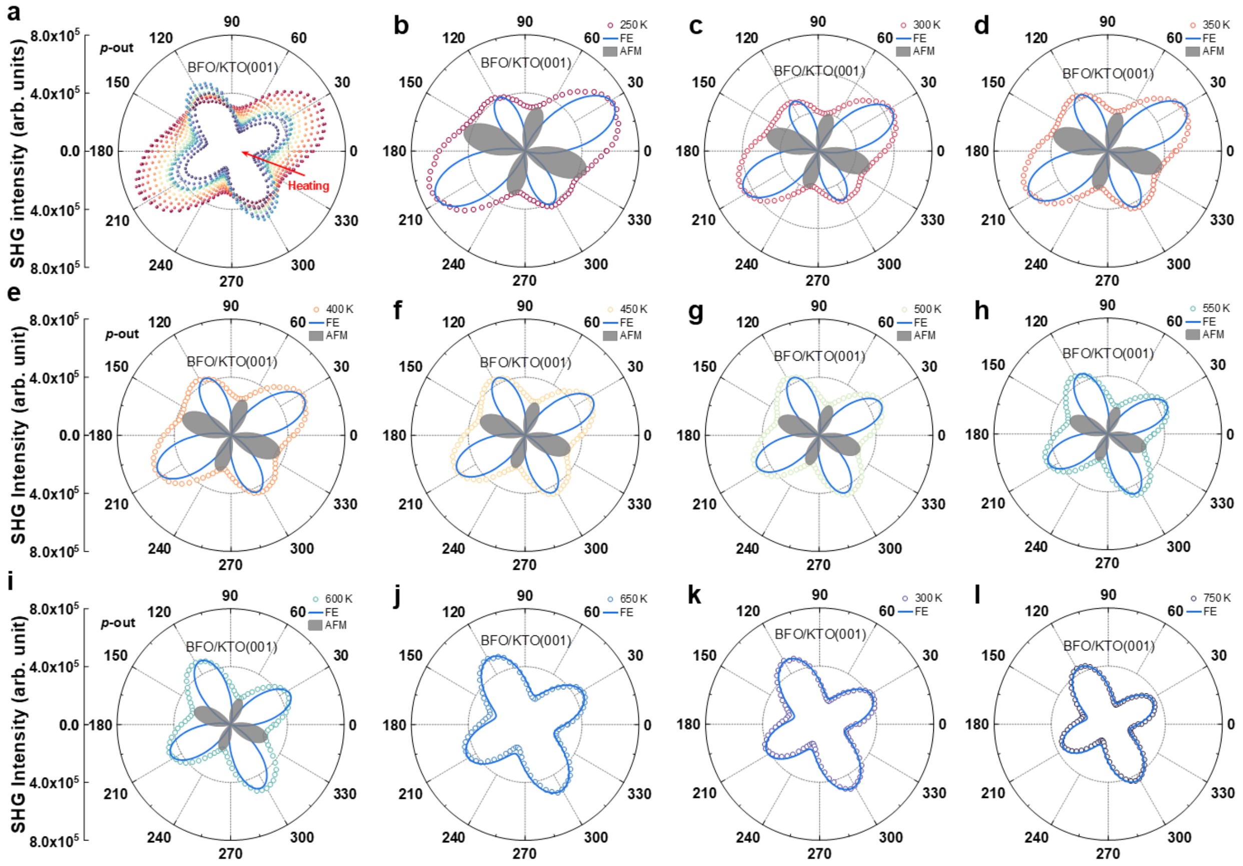

Supplementary Figure 8 | a RA-SHG results of BFO/KTO films at different temperatures, the scattered points are the experimental data at different temperatures. **b-i** RA-SHG results and analyses of BFO/KTO films below T_N (about 646 K for BFO/KTO films), where both the antiferromagnetic order and the ferroelectric order contribute to the SHG signal, leading to the asymmetry of the RA-SHG patterns. **j-l** RA-SHG results and analyses of BFO/KTO films above T_N, where contribution from the antiferromagnetic order disappears and only the ferroelectric order contributes to the SHG signal. The hollow dots are the experimental data, the blue lines represent the contribution from the ferroelectric order, and the gray shadows represent the contribution from the antiferromagnetic order.

9. First-principles calculation results of the crystal structures of BFO with different strains

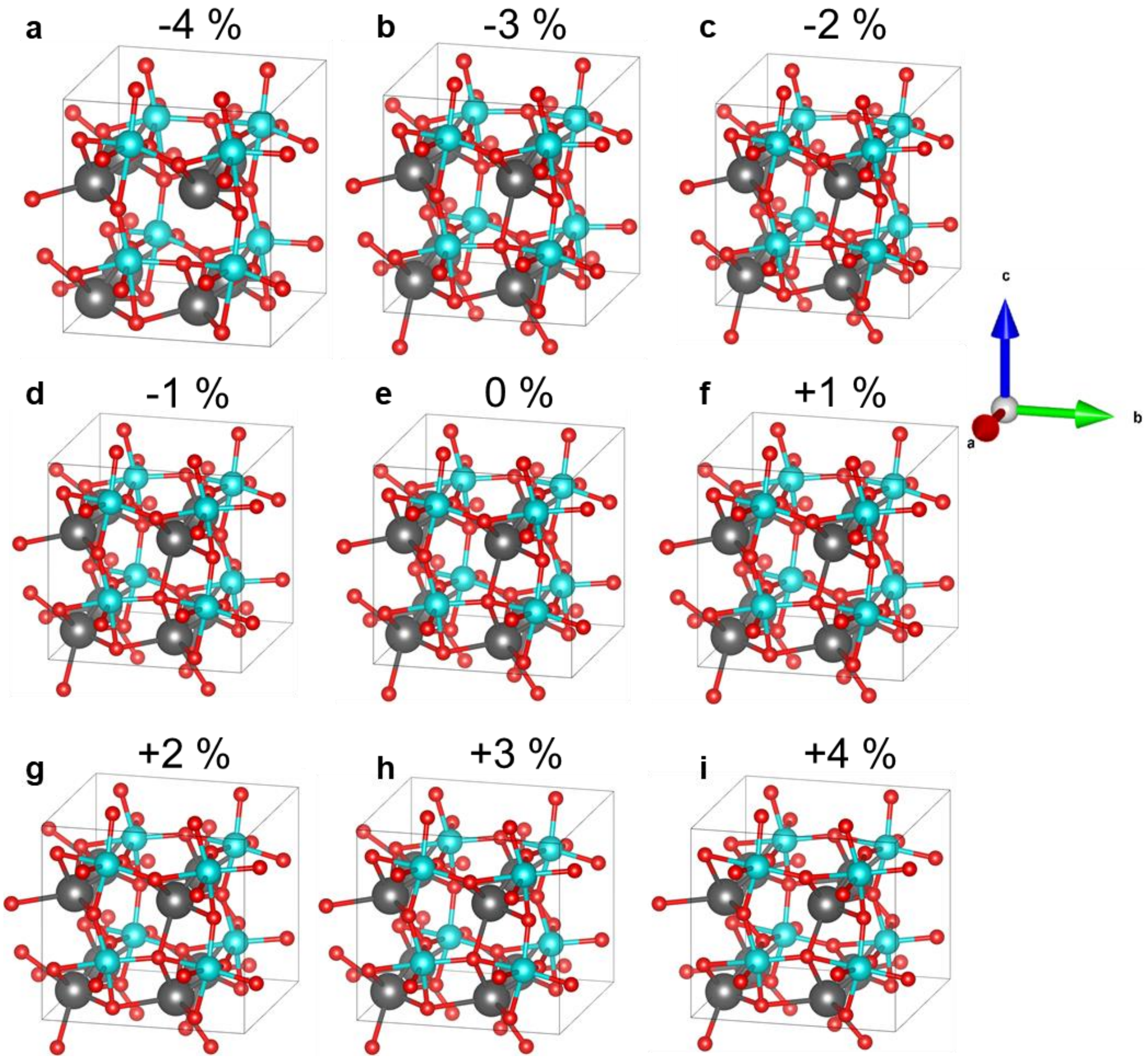

Supplementary Figure 9 | a-i Crystal structures of BFO with strains of -4%, -3%, -2%, -1%, 0%, +1%, +2%, +3%, and +4%, respectively.

10. $(1\bar{1}0)$ plane-view of the BFO crystal structure with different strains

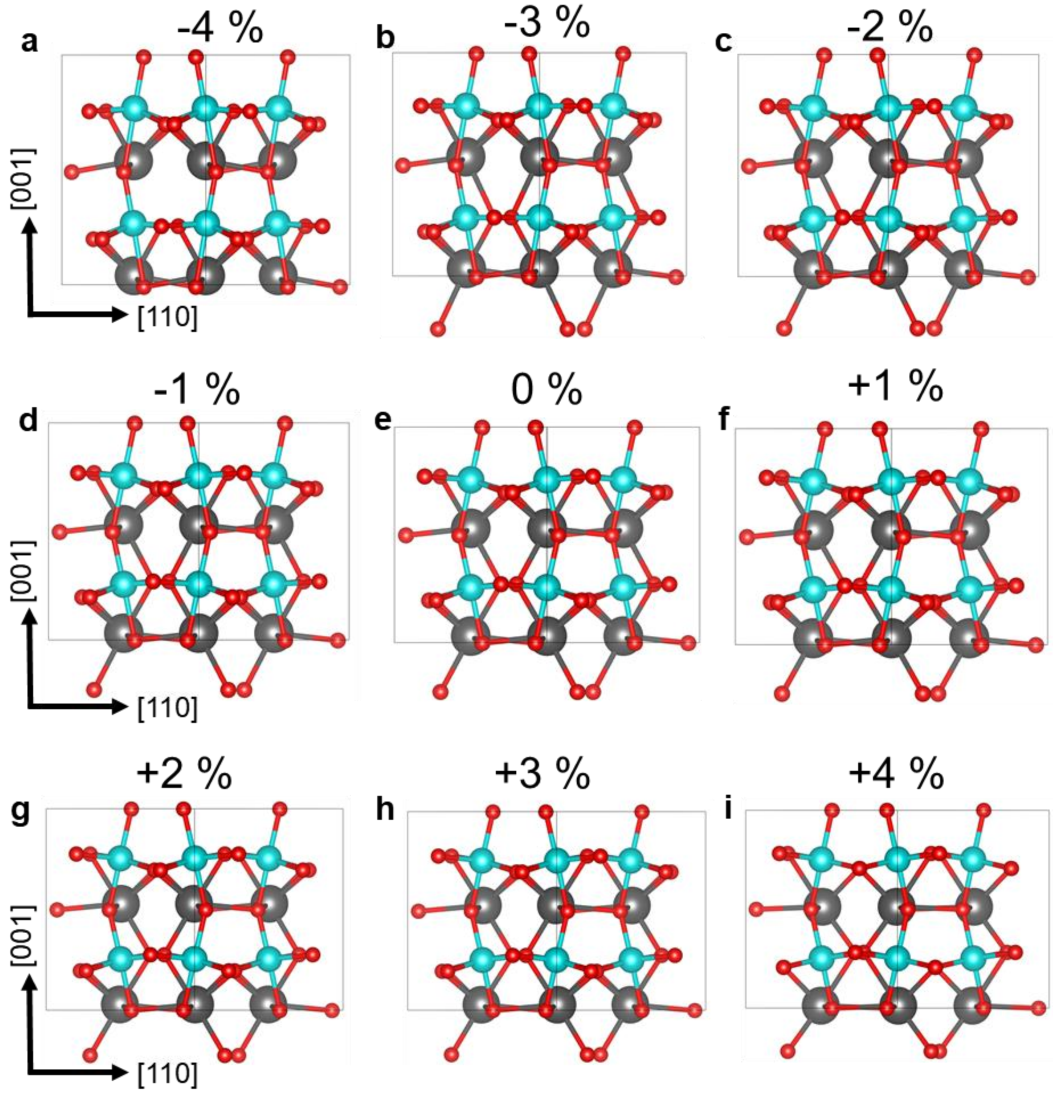

Supplementary Figure 10 | a-i $(1\bar{1}0)$ plane- view of the BFO crystal structure with strains of -4%, -3%, -2%, -1%, 0%, +1 %, +2%, +3%, and +4%, respectively.

11. Functional forms of electric dipole SHG for crystallographic 4*mm* and magnetic point-group *m*

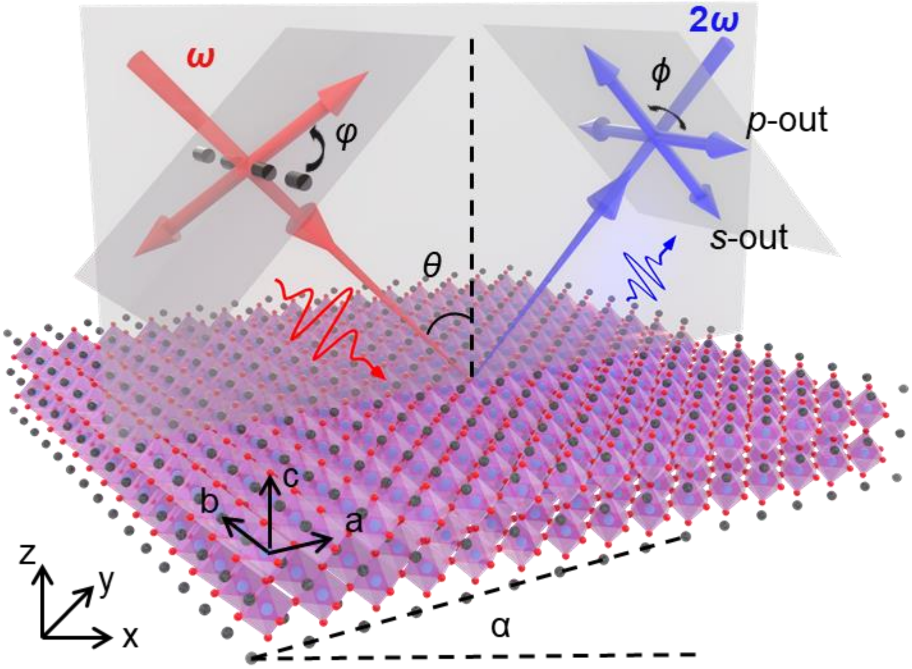

Supplementary Figure 11 | Schematic diagram of the rotating-polarization-based RA-SHG setups.

The second-order nonlinear optical process can be expressed as: $\boldsymbol{P}(2\omega) = \chi^{(2)} : \boldsymbol{E}(\omega)\boldsymbol{E}(\omega)$, where $\chi^{(2)}$ is the second-order nonlinear optical susceptibility tensor of the three-rank, and $E(\omega)$ is the incident optical electric field of the femtosecond laser with a frequency ω[1, 2]. In general, the three-rank electric dipole (ED) term $\chi_{ED}^{(2)}(\omega)$ plays a dominant role in the contribution of the SHG signal, and $\chi_{ED}^{(2)}(\omega)$ can only be produced in non-centrosymmetric medium (such as ferroelectric materials, surfaces, and interfaces)[3, 4]. For systems with broken spatial-inversion symmetry, information related to the crystal structure symmetry and phase transitions (such as crystal point groups and phase transition temperatures) can be analyzed from the results obtained from wide-temperature-range RA-SHG measurements[5].

For a ferroelectric crystalline material with antiferromagnetic order (such as multiferroic BFO), $\chi^{(2)}$ can be expressed as: $\chi_{ED}^{(2)} = \varepsilon_0\left(\chi^{(i)} + \chi^{(c)}\right)$, where the time-invariant tensor $\chi^{(i)}$ and the time-noninvariant tensor $\chi^{(c)}$ represent the crystallographic (spatial-inversion symmetry breaking) and antiferromagnetic order structure (time-reversal symmetry breaking), respectively[6, 7]. With an approximation that considers only the electric dipole, the SHG source term $S(2\omega)$ is

related to the light-induced nonlinear polarization $\boldsymbol{P}(2\omega)$ in the following way: $S(2\omega) = \mu_0 \frac{\partial^2 \boldsymbol{P}(2\omega)}{\partial t^2}$, where $\boldsymbol{P}(2\omega) = \varepsilon_0\left(\chi^{(i)} + \chi^{(c)}\right) : \boldsymbol{E}(\omega)\boldsymbol{E}(\omega)$. The SHG signal measured experimentally is the intensity $I^{2\omega}(\varphi)$ of the 2ω light, and its relationship with $\boldsymbol{P}$ is:

$$I^{2\omega}(\varphi) \propto |\boldsymbol{P}(2\omega)|^2 \propto \left|\hat{e}_{i'}(2\omega)\chi^{ED}_{i'j'k'}\hat{e}_{j'}(\omega)\hat{e}_{j'}(\omega)\right|^2. \tag{1}$$

To analyze SHG experimental data, the second-order nonlinear optical susceptibility tensor should be represented in the laboratory coordinate system (*x*, *y*, *z*). We generally try to make the laboratory coordinate system parallel to the crystal coordinate system. Here, the incident angle θ is 45°, as shown in the schematic diagram of Supplementary Fig. 12. By rotating the polarization angle φ of the incident light and fixing the polarization of the reflection light as p ($\phi = 0°$, parallel to the plane of incidence) or s ($\phi = 90°$, perpendicular to the plane of incidence), the variation of the SHG intensity can be obtained. For non-centrosymmetric crystal 4*mm* point groups, the time-invariant SHG susceptibility tensor $\chi^{(i)}$ is:

$$\chi^{(i)} = \begin{pmatrix} 0 & 0 & 0 & 0 & \chi^{(i)}_{15} & 0 \\ 0 & 0 & 0 & \chi^{(i)}_{15} & 0 & 0 \\ \chi^{(i)}_{31} & \chi^{(i)}_{31} & \chi^{(i)}_{33} & 0 & 0 & 0 \end{pmatrix}, \tag{2}$$

for *p*-out configuration, so $\phi = 0°$. Substituting the time-invariant SHG susceptibility tensor $\chi^{(i)}$ into $\boldsymbol{P}(2\omega)$, we have:

$$\boldsymbol{P}^{(i)}_{p-out}(2\omega) = Acos^2\varphi + Bsin^2\varphi, \tag{3}$$

$$A = \frac{2\sqrt{2}L^{2\omega}_{yy}L^{\omega}_{yy}L^{\omega}_{zz}\chi^{(i)}_{15} - \sqrt{2}(L^{\omega}_{yy})^2 L^{2\omega}_{zz}\chi^{(i)}_{31} - \sqrt{2}(L^{\omega}_{zz})^2 L^{2\omega}_{zz}\chi^{(i)}_{33}}{4}, \tag{4}$$

$$B = -\frac{\sqrt{2}(L^{\omega}_{xx})^2 L^{2\omega}_{zz}\chi^{(i)}_{31}}{2}, \tag{5}$$

in which L_{xx}, L_{yy}, and L_{zz} are the diagonal elements of the transmitted Fresnel factor L in fundamental and second harmonic optical materials. Considering $I^{2\omega}(\varphi) \propto |\boldsymbol{P}(2\omega)|^2$, we obtained:

$$I^{(i)}_{p-out}(\varphi) \propto (Acos^2\varphi + Bsin^2\varphi)^2. \tag{6}$$

Below T_N, the existence of the antiferromagnetic order in BFO films breaks the time-reversal symmetry. As the non-centrosymmetric magnetic order also breaks the spatial-inversion symmetry, the magnetization-induced ED-SHG is produced. With magnetic point-group *m*, the time-noninvariant SHG susceptibility tensor $\chi^{(c)}$ is[7]:

$$\chi^{(c)} = \begin{pmatrix} \chi_{11}^{(c)} & \chi_{12}^{(c)} & \chi_{13}^{(c)} & 0 & \chi_{15}^{(c)} & 0 \\ 0 & 0 & 0 & \chi_{24}^{(c)} & 0 & \chi_{12}^{(c)} \\ \chi_{15}^{(c)} & \chi_{24}^{(c)} & \chi_{33}^{(c)} & 0 & \chi_{13}^{(c)} & 0 \end{pmatrix}. \tag{7}$$

Similarly, substituting the time-noninvariant SHG susceptibility tensor $\chi^{(c)}$, we have:

$$\boldsymbol{P}_{p-out}^{(c)}(2\omega) = Ccos^2\varphi + Dsin^2\varphi + Esin2\varphi, \tag{8}$$

$$C = \frac{2\sqrt{2}L_{yy}^{2\omega}L_{yy}^{\omega}L_{zz}^{\omega}\chi_{24}^{(c)} - \sqrt{2}(L_{yy}^{\omega})^2 L_{zz}^{2\omega}\chi_{24}^{(c)} - \sqrt{2}(L_{zz}^{\omega})^2 L_{zz}^{2\omega}\chi_{33}^{(c)}}{4}, \tag{9}$$

$$D = -\frac{\sqrt{2}(L_{xx}^{\omega})^2 L_{zz}^{2\omega}\chi_{15}^{(c)}}{2}, \tag{10}$$

$$E = \frac{L_{yy}^{2\omega}L_{xx}^{\omega}L_{yy}^{\omega}\chi_{12}^{(c)} - L_{zz}^{2\omega}L_{xx}^{\omega}L_{zz}^{\omega}\chi_{13}^{(c)}}{2}. \tag{11}$$

Considering $I^{2\omega}(\varphi) \propto |\boldsymbol{P}(2\omega)|^2$, we obtained:

$$I_{p-out}^{(c)}(2\omega) \propto (Ccos^2\varphi + Dsin^2\varphi + Esin2\varphi)^2. \tag{12}$$

References

1. Zhang Y, *et al.* Doping-Induced Second-Harmonic Generation in Centrosymmetric Graphene from Quadrupole Response. *Phys Rev Lett* **122**, 047401 (2019).
2. Fiebig M, Pavlov VV, Pisarev RV. Second-harmonic generation as a tool for studying electronic and magnetic structures of crystals: review. *Journal of the Optical Society of America B* **22**, 96-118 (2005).
3. Shen Y-R. *The principles of nonlinear optics* (1984).
4. Fiebig M, Fröhlich D, Krichevtsov BB, Pisarev RV. Second Harmonic Generation and Magnetic-Dipole-Electric-Dipole Interference in Antiferromagnetic Cr_2O_3. *Phys Rev Lett* **73**, 2127-2130 (1994).
5. Xu S, *et al.* Magnetoelectric coupling in multiferroics probed by optical second harmonic generation. *Nature Communications* **14**, 2274 (2023).
6. Birss RR. *Symmetry and magnetism*. North-Holland Publishing Company (1964).
7. Chauleau JY, Haltz E, Carrétéro C, Fusil S, Viret M. Multi-stimuli manipulation of antiferromagnetic domains assessed by second-harmonic imaging. *Nat Mater* **16**, 803-807 (2017).
